# DDRGK1, a crucial player of ufmylation system, is indispensable for autophagic degradation by regulating lysosomal function

**DOI:** 10.1038/s41419-021-03694-9

**Published:** 2021-04-20

**Authors:** Yan Cao, Rongyang Li, Ming Shen, Chengyu Li, Yan Zou, Qiang Jiang, Shuo Liu, Chunwan Lu, Honglin Li, Honglin Liu, Yafei Cai

**Affiliations:** 1 Department of Animal Genetics, Breeding and Reproduction, College of Animal Science and Technology, Nanjing Agricultural University, 210095 Nanjing, China; 2 School of life sciences, Tianjin University, 300072 Tianjin, China; 3 Department of Biochemistry and Molecular Biology, Medical College of Georgia, Augusta University, Augusta, GA 30912 USA

**Keywords:** Macroautophagy, Apoptosis, Endoplasmic reticulum

## Abstract

DDRGK domain-containing protein 1 (DDRGK1) is an important component of the newly discovered ufmylation system and its absence has been reported to induce extensive endoplasmic reticulum (ER) stress. Recently, emerging evidence indicates that the ufmylation system is correlated with autophagy, although the exact mechanism remains largely unknown. To explore the regulation mechanism of DDRGK1 on autophagy, in this study, we established an immortalized mouse embryonic fibroblast (MEF) cell lines harvested from the DDRGK1^F/F^:ROSA26-CreERT2 mice, in which DDRGK1 depletion can be induced by 4-hydroxytamoxifen (4-OHT) treatment. Here, we show that DDRGK1 deficiency in MEFs has a dual effect on autophagy, which leads to a significant accumulation of autophagosomes. On one hand, it promotes autophagy induction by impairing mTOR signaling; on the other hand, it blocks autophagy degradation by inhibiting autophagosome–lysosome fusion. This dual effect of DDRGK1 depletion on autophagy ultimately aggravates apoptosis in MEFs. Further studies reveal that DDRGK1 loss is correlated with suppressed lysosomal function, including impaired Cathepsin D (CTSD) expression, aberrant lysosomal pH, and v-ATPase accumulation, which might be a potential trigger for impairment in autophagy process. Hence, this study confirms a crucial role of DDRGK1 as an autophagy regulator by controlling lysosomal function. It may provide a theoretical basis for the treatment strategies of various physiological diseases caused by DDRGK1 deficiency.

## Introduction

The ubiquitin-fold modifier 1 (Ufm1) conjugation system is a novel ubiquitin-like modification system that consists of a set of ubiquitin-like proteins (UBLs), including Ufm1, Ufm1-activating E1 enzyme (Uba5), Ufm1-conjugating E2 enzyme (Ufc1), and Ufm1-specific E3 ligase (Ufl1, also known as RACD, NLBP and Maxer)^1–3^. DDRGK domain-containing protein 1 (DDRGK1, also known as C20orf116, and Dashurin), another critical component of the Ufm1 conjugation system, was found as a Ufm1 target whose ufmylation was conducted by Ufl1 (ref. ^2^). It contains a proteasome component (PCI) domain that is generally associated with protein interactions and a putative signal peptide at its N-terminus that contributes to its endoplasmic reticulum (ER) anchoring^4^. Studies show that DDRGK1, together with several other Ufm1 system components Ufl1, C53/LZAP (Cdk5 activator-binding protein Cdk5rap3)^4,5^ and Ufm1, forms a large complex that is anchored at the ER and each of these proteins is indispensable for maintaining ER homeostasis^2,6,7^. Recent studies have shown that DDRGK1 deficiency can cause a variety of physiological and pathological diseases, including hematopoietic dysfunction^8^, impaired intestinal homeostasis^9^, and compromised plasma cell development^10^. This evidence indicates a critical role of DDRGK1 in maintaining normal physiological processes, though the underlying mechanism is poorly understood.

Macrophagy (hereafter referred to as autophagy), an evolutionary conserved biological process in eukaryotes, is considered to maintain cell homeostasis by engulfing the cytoplasmic proteins or damaged organelles in double-membrane-bound structures, known as autophagosomes, and then deliver them to the lysosome for degradation^11,12^. Autophagy is rapidly induced by nutrient deprivation^13^, oxidative stress^14^, hypoxia^15,16^, ER stress^17,18^, and other cellular stresses and diseases^19,20^. The stimulation of autophagy is a complex dynamic process, which requires plenty of autophagy-related genes (ATGs), including two vital ubiquitin-like conjugation systems^21^, Atg5–Atg12–Atg16L1 complex and microtubule-associated protein light chain 3 (LC3, a homolog of yeast Atg8)^22^. Atg7 is considered to be involved in both of these two conjugation systems, and its deficiency may impair the autophagy induction. Autophagosome–lysosome fusion is another essential step in autophagy process. It drives autophagic degradation, which is generally achieved by soluble *N*-ethylmaleimide-sensitive factor attachment protein receptor proteins (SNAREs). Especially the STX17 (syntaxin 17)–SNAP29 (synaptosome associated protein 29)–VAMP8 (vesicle-associated membrane protein 8) complex is widely considered to mediate autophagosome–lysosome fusion^23,24^.

As emerging evidence indicates that ER stress-induced unfolded protein reaction promotes autophagy stimulation^25–28^, it seems that the DDRGK1 is implicated in autophagy regulation. In fact, a previous study had shown that Ufl1 loss leads to impaired autophagic degradation^29^, providing evidence for the involvement of ufmylation system in autophagy regulation. However, it remains elusive whether DDRGK1 is required for this process.

Therefore, in this study, immortalized mouse embryonic fibroblast (MEF) cell lines harvested from the DDRGK1^F/F^:ROSA26-CreERT2 mice (with thorough DDRGK1 knockout efficiency) were used as a research model to investigate the role of DDRGK1 in autophagy regulation. Here, for the first time, we demonstrated that DDRGK1 deletion suppressed the autophagosome–lysosome fusion, thus leading to an impaired autophagic degradation and increased apoptosis in MEFs. Collectively, our findings reveal a DDRGK1-dependent mechanism in the regulation of autophagic flux.

## Results

### Result 1: 4-OHT-induced DDRGK1 deficiency promotes apoptosis in MEFs

**Fig. 1:**
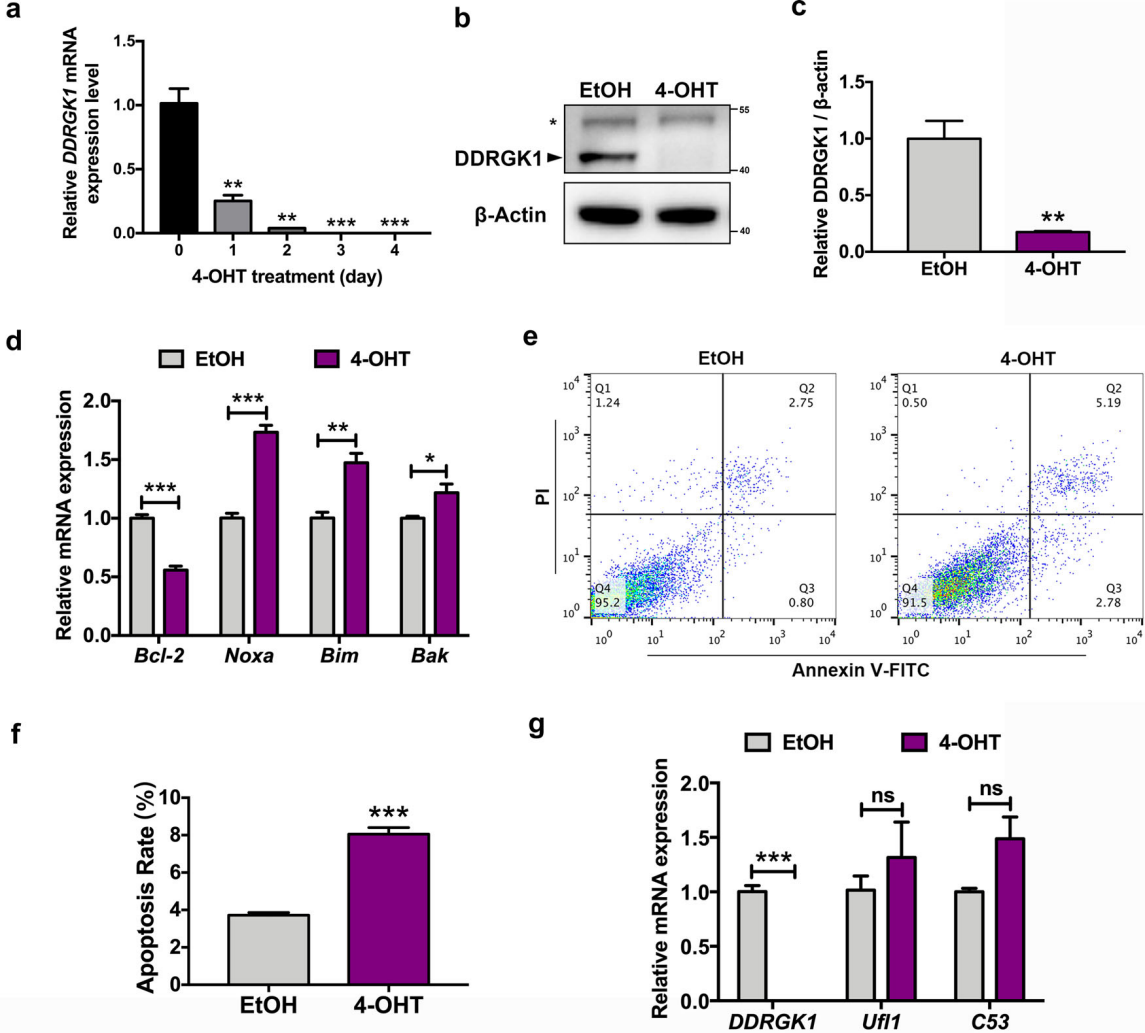
DDRGK1 loss leads to elevated apoptosis in MEFs. **a** MEFs were treated with 4-OHT (2 μM) for 0, 1, 2, 3, and 4 days. The indicated samples were then collected for qRT-PCR analysis to determine the transcriptional levels of *DDRGK1*. Data were shown as mean ± SEM, *n* = 3. **P < 0.01; ***P < 0.001. **b** MEFs were treated with EtOH or 4-OHT (2 μM) for 4 days. Cell lysates were collected to determine the DDRGK1 levels using western blotting. Asterisk indicates nonspecific bands. **c** Quantification of DDRGK1 levels (normalized to β-actin) in EtOH- and 4-OHT-treated cells. Data were shown as mean ± SEM, *n* = 3. **P < 0.01. **d** MEFs were cultured in EtOH or 4-OHT (2 μM) for 4 days. qRT-PCR assay was performed to detect the mRNA levels of apoptosis-related genes, including *Bcl-2*, *Noxa*, *Bim*, and *Bak*. Data were shown as mean±SEM, *n* = 3. *P < 0.05; **P < 0.01; ***P < 0.001. **e** Flow cytometry analysis of apoptosis in MEFs incubated with EtOH or 4-OHT for 4 days. **f** Quantification of apoptosis rate in control and DDRGK1-deficient MEFs. Data were shown as mean ± SEM, *n* = 3. ***P < 0.001. **g** MEFs treated with EtOH or 2 μM 4-OHT for 4 days were then analyzed by qRT-PCR to determine the mRNA levels of *DDRGK1*, *Ufl1*, and *C53/LZAP*. Data were presented as mean ± SEM, *n* = 3. ***P < 0.001; ns not significant, *P* > 0.05.

To investigate the function of DDRGK1, immortalized MEFs derived from the DDRGK1^F/F^:ROSA26-CreERT2 mice^8^ were treated with 4-hydroxytamoxifen (4-OHT) to induce the deficiency of DDRGK1. As is shown in Fig. 1a, DDRGK1 mRNA was significantly decreased in a time-dependent manner and was almost undetectable on the third and fourth days. Consistently, >80% of DDRGK1 protein was depleted after treating with 4-OHT for 4 days (Fig. 1b, c). Our previous study suggests that DDRGK1 deletion leads to cell death of hematopoietic stem cells (HSCs)/progenitor cells^8^. Likewise, we found that DDRGK1 deficiency led to cell apoptosis, for a subset of proapoptotic genes, including *Noxa*, *Bim*, and *Bax* were significantly upregulated and *Bcl-2*, an antiapoptotic gene were remarkably downregulated in MEFs (Fig. 1d). Furthermore, flow cytometry analysis showed a significant increased apoptosis in 4-OHT group compared with the control group (Fig. 1e, f). We also measured the mRNA level of *Ufl1* and *C53/LZAP*, as they are other two novel components of Ufm1 system, which have a crosstalk with DDRGK1 (ref. ^4^). Interestingly, the mRNA levels of *Ufl1* and *C53/LZAP* were not affected by *DDRGK1* deletion (Fig. 1g), suggesting the aforementioned results were indeed caused by DDRGK1 deletion. Taken together, these data suggest that 4-OHT mediates a sufficient deletion of DDRGK1, and DDRGK1 is required for maintaining the normal physiological balance in MEFs.

### Result 2: DDRGK1 deficiency is involve in autophagy–lysosome-related regulation

Although DDRGK1 has been reported to regulate numerous physiological processes, ranging from HSC development^8^, plasma cell development^10^ to intestinal inflammation^9^, the underlying mechanism remains poorly understood. Thus, we performed iTRAQ-based liquid chromatography–mass spectrometry (LC–MS) to identify differentially expressed proteins between the control and DDRGK1-deleted MEFs. The hierarchical clustering analysis showed a high biological reproducibility both in control and 4-OHT groups (Fig. S1a and Table S3).

**Fig. 2:**
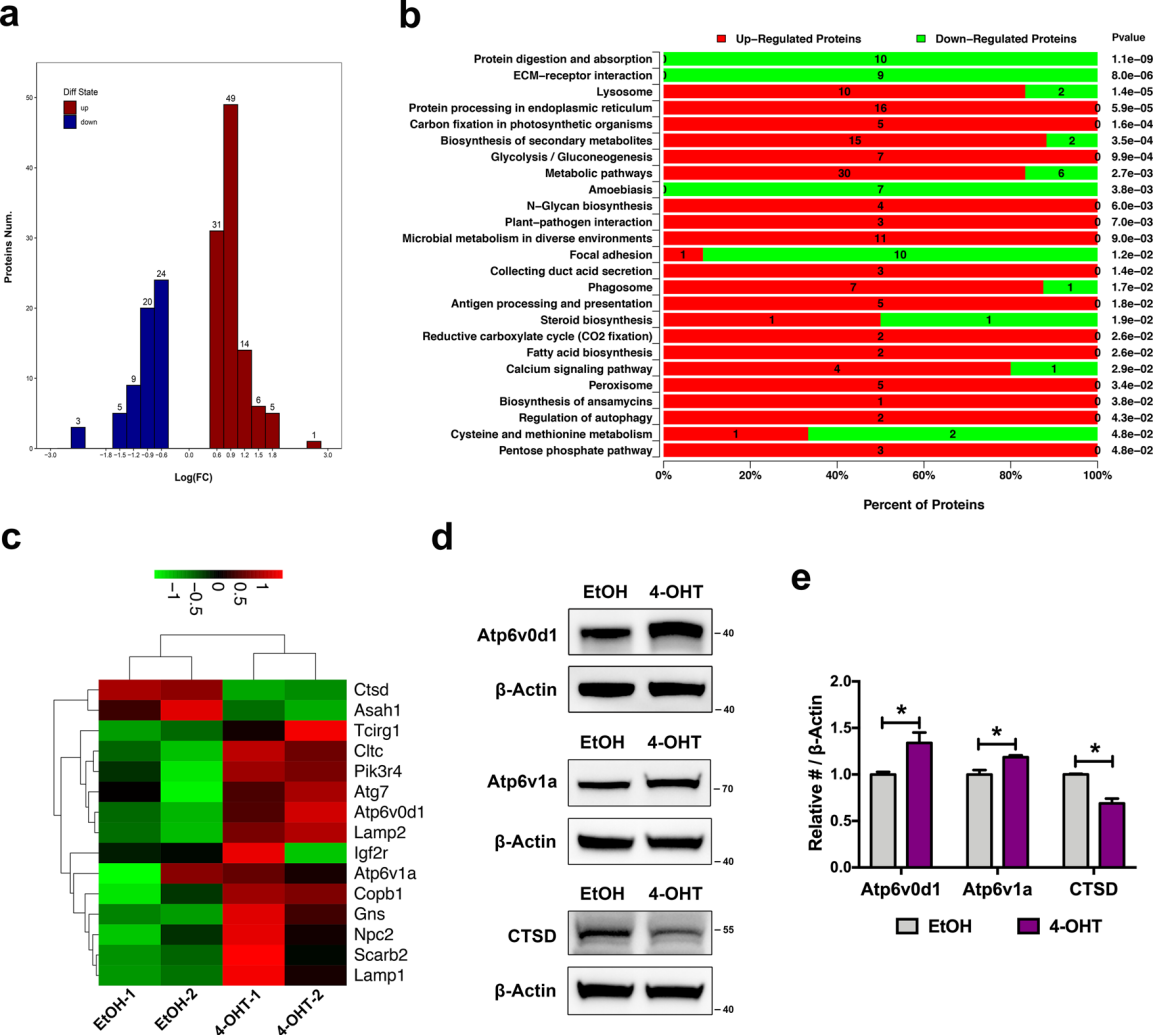
iTRAQ-proteomics and bioinformatics analyses of DDRGK1-deleted MEFs. **a** Distribution of differential expressed proteins between DDRGK1-deleted and the control groups detected by LC–MS. The number of changed proteins in each category of log(FC) is indicated above each bar. **b** Specific significantly enriched KEGG pathways in 4-OHT- or EtOH-treated groups. Number of upregulated and downregulated proteins is shown on the bar plot. The *P* values is shown for each pathway based on hypergeometric distribution. **c** Expression patterns of proteins enriched in lysosome- and autophagy-related pathways. **d** Western blot analysis for indicated proteins. MEFs treated with EtOH or 4-OHT for 4 days were then subjected to western blotting to validate the expression changes of selected representatives—Atp6v0d1, Atp6v1a, and CTSD. **e** Quantificational expressions of Atp6v0d1, Atp6v1a, and CTSD protein standardized by β-actin expression. Data represent mean ± SEM, *n* = 3. *P < 0.05.

A total of 5092 proteins were detected, of which 167 were differentially expressed (Fig. 2a and Table S3). Consistent with what we expected, a large number of differential expressed proteins were enriched in KEGG pathways related to ER homeostasis and protein processing, such as “protein processing in ER” and “protein digestion and absorption” terms (Fig. 2b and Table S4; Fig. S1b and Table S6). Importantly, a considerable differential expressed proteins were enriched in lysosome-related pathways, including “lysosome”, “regulation of autophagy”, and “collecting duct acid secretion” terms (Fig. 2b and Table S4; Fig. S1b and Table S6), suggesting that DDRGK1 might participate in autophagy–lysosome-related degradation process. Therefore, we further analyzed the 15 differentially expressed proteins in these three terms and found that a majority of proteins were upregulated with only CTSD and ASAH1 downregulated (Fig. 2c and Table S5). Changes were validated by western blot analysis, which showed a significant increase in Atp6v0d1 and Atp6v1a, and a decrease in CTSD (Fig. 2d, e). These data suggest that DDRGK1 have a potential regulation on autophagy–lysosome-related pathway.

### Result 3: DDRGK1 deletion causes a significant accumulation of autophagosomes in vitro and in vivo

**Fig. 3:**
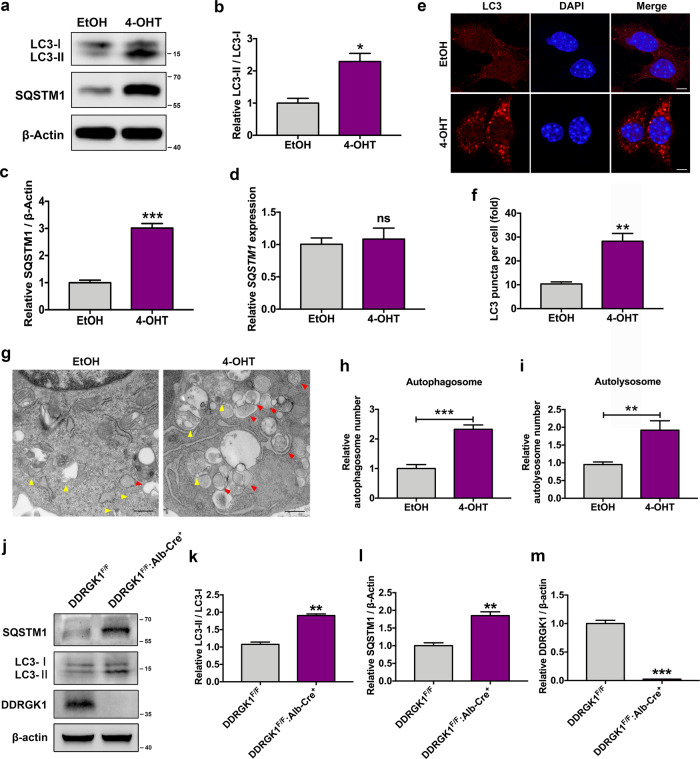
DDRGK1 loss accelerates the accumulation of autophagosomes. **a** Western blot analysis of LC3 and SQSTM1 protein in cell lysates from control and DDRGK1-deficient MEFs. **b**, **c** Quantification of conversion of LC3-I to LC3-II and SQSTM1 expression standardized by β-actin level. Data represent mean ± SEM; *n* = 3. *P < 0.05; ***P < 0.001. **d** The mRNA level of *SQSTM1* determined by qRT-PCR analysis. Data represent mean ± SEM; *n* = 3. ns not significant, *P* > 0.05. **e** Immunofluorescence staining of endogenous LC3 protein in EtOH- and 4-OHT-treated MEFs. Each red puncta represents a autophagosome accumulated in the cytoplasm of MEFs. Laser confocal-scanning microscopy was employed to observe the fluorescent puncta in MEFs. Bar, 10 μm. **f** Quantification of the punctate LC3 per cell. Data represent mean ± SEM; *n* = 3. **P < 0.01. **g** TEM images of autophagic vesicle in control and DDRGK1-deleted MEFs. Red arrowheads represent autophagosomes and yellow ones represent autolysosomes. Bar, 0.5 μm. **h**, **i** Quantification of autophagic vesicle numbers per imaging area. Data represent mean ± SEM; *n* = 3. **P < 0.01; ***P < 0.001. **j** Western blot analysis of LC3, SQSTM1, and DDRGK1 proteins in liver tissues of wild-type and DDRGK1-deficient mice. **k**–**m** Quantification of conversion of LC3-I to LC3-II (**k**), SQSTM1 (**l**), and DDRGK1 (**m**) expression standardized by β-actin expression. Data represent mean ± SEM; *n* = 3. **P < 0.01; ***P < 0.001.

To validate the effects of DDRGK1 on MEF autophagy, we first examined the expression of LC3 protein, a typical biomarker of autophagy, and SQSTM1, a substrate of autophagic degradation, which would be degraded by autolysosome during autophagic degradation^30,31^. As is shown in Fig. 3a–d, both conversion of LC3-I to LC3-II and SQSTM1 expression was significantly promoted, while *SQSTM1* transcript level was not affected in MEFs after treating with 4-OHT. Accordingly, immunofluorescence assay with LC3 staining showed an elevated number of LC3-positive puncta in DDRGK1-deleted cells (Fig. 3e, f), suggesting an accumulation of autophagosomes. Using transmission electron microscopy (TEM), we detected a significant accumulation of autophagic vesicles (both autophagosomes and autolysosomes). These data further confirm that DDRGK1 deletion facilitates accumulation of autophagosomes in MEFs (Fig. 3g–i).

To determine if the regulation of DDRGK1 on autophagy was conserved in vivo, we detected the LC3 and SQSTM1 level in liver tissues of wild-type and DDRGK1^F/F^: ALB-cre^+^ mice. Likewise, DDRGK1 loss led to a dramatic accumulation of SQSTM1 and LC3-II conversion in DDRGK1-deleted liver tissues (Fig. 3j–m), indicating the function of DDRGK1 in autophagy regulation is conserved in vivo. Taken together, these data suggest that DDRGK1 deletion lead to a significant accumulation of autophagosomes both in vitro and in vivo.

**Fig. 4:**
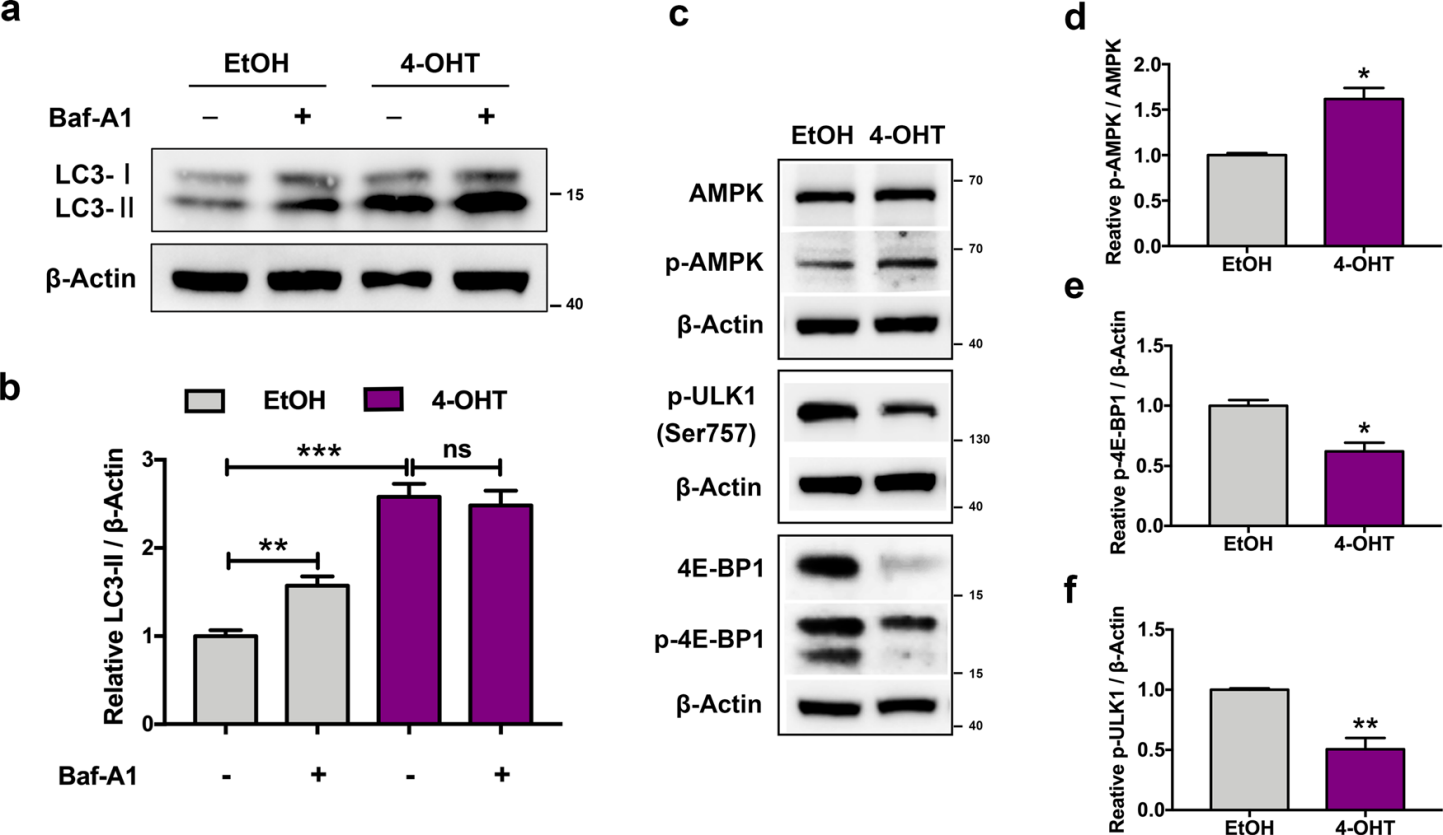
DDRGK1 deficiency promotes autophagy induction via impairing mTOR signaling. **a** Western blot analysis of LC3 protein in control and DDRGK1-deficient MEFs treated with or without 10 nM Baf-A1. **b** Quantification of LC3-II level that normalized to β-actin content. Data represent mean ± SEM; *n* = 3. **P < 0.01; ***P < 0.001; ns not significant, *P* > 0.05. **c** Western blot for phosphorylation of AMPK, ULK1, and 4E-BP1 in cell lysates from EtOH- and 4-OHT-treated MEFs. **d**–**f** Quantification of p-AMPK/AMPK (**d**), p-ULK1/β-Actin (**e**), and p-4E-BP1/4E-BP1 (**f**) ratios, respectively. Data were presented as mean ± SEM; *n* = 3. *P < 0.05; **P < 0.01.

To further assess whether the accumulation of autophagosomes is caused by enhanced autophagosome formation or attenuated autolysosome degradation, we next detected LC3 expression in the presence of bafilomycin A1 (Baf-A1), an autophagy inhibitor that blocks fusion of autophagosomes with lysosomes. Compared with the control group, the LC3-II level was significantly increased after Baf-A1 addition. However, the addition of Baf-A1 brought no further change in LC3-II level in 4-OHT-treated groups, indicating a potential suppression of autophagic degradation during DDRGK1 deficiency. Notably, on the basis of Baf-A1 supplement (serves as an inhibition of autophagic degradation), the expression of LC3-II was further dramatically elevated in DDRGK1-deleted group when compared with the control group (Fig. 4a, b), suggesting that DDRGK1 deficiency might concomitantly promote autophagosome formation in addition to suppressing autophagic degradation.

### Result 4: autophagy induction is associated with an impaired mTOR signaling in DDRGK1-deficient cells

mTORC1 is an important regulator of autophagy, whose dephosphorylation promotes autophagy induction^32,33^. To verify if DDRGK1 deletion promotes autophagy initiation, we tested the activity of mTORC1 by detecting the phosphorylation status of mTORC1 targets, ULK1 (refs. ^34,35^) and 4E-BP1. As is shown in Fig. 4c, e, f, DDRGK1 deletion suppressed the phosphorylation of ULK1 (Ser757) and 4E-BP1, indicating a decreased activity of mTORC1. Moreover, the increased phosphorylation of AMPK (another upstream regulator of autophagy)^36,37^ in DDRGK1-deficient group further confirmed the enhanced autophagy induction (Fig. 4c, d). These data suggest that DDRGK1 deficiency promotes autophagy induction by controlling the activity of autophagy upstream kinases.

### Result 5: DDRGK1 is indispensable in maintaining autophagic flux

**Fig. 5:**
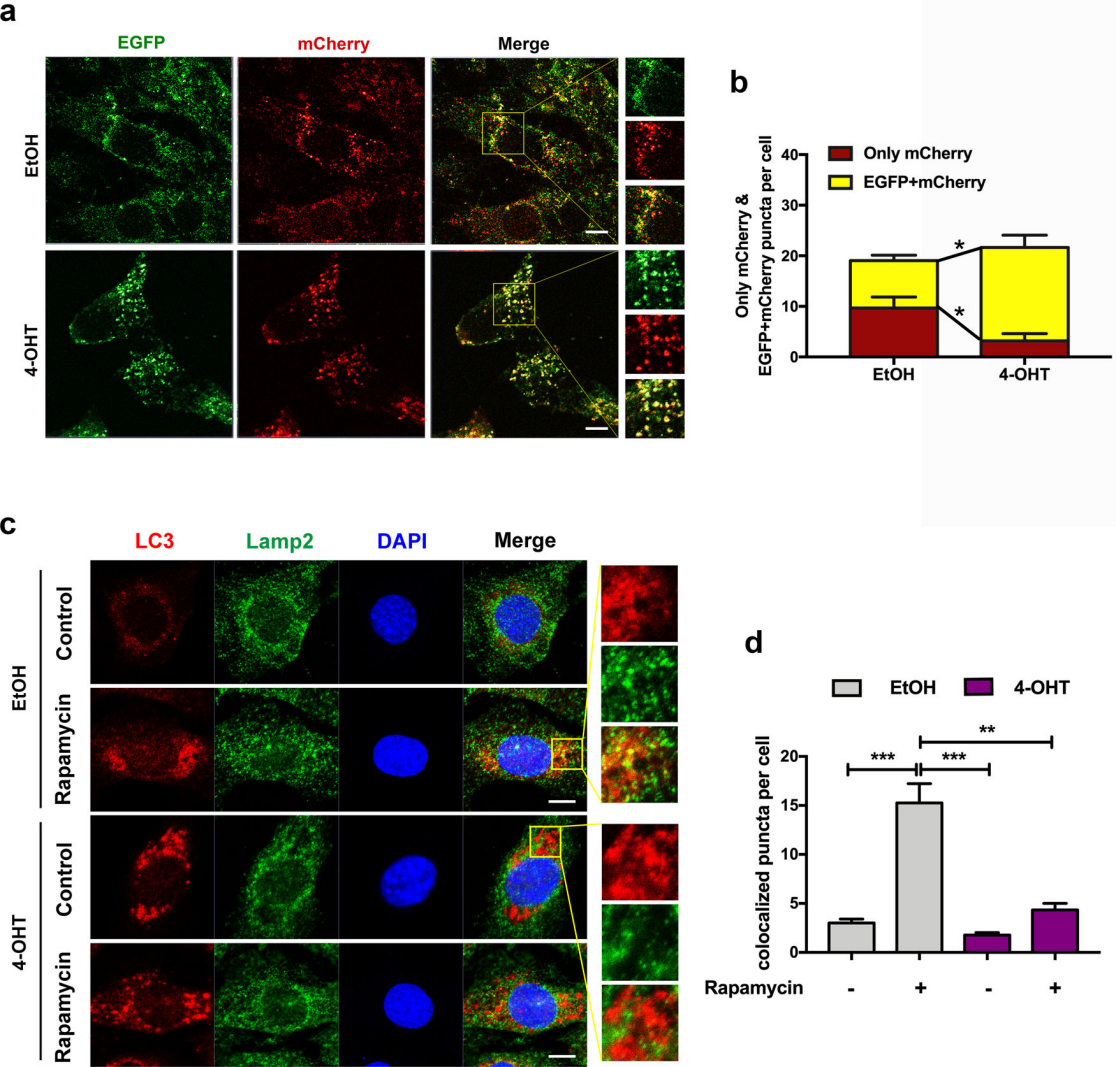
DDRGK1 loss impairs autophagic flux. **a** Representative images of control and DDRGK1-deleted MEFs transfected with mCherry-EGFP-LC3 plasmid for 36 h. Enlarged images of the yellow box were indicated on the right side. Laser confocal-scanning microscopy was employed to observe the fluorescent puncta. Bar, 10 μm. **b** Quantification of only mCherry-labeled and EGFP + mCherry-labeled puncta. Data represent mean ± SEM; *n* = 3. *P < 0.05. **c** Colocalization of endogenous LC3 and LAMP2 proteins in control and DDRGK1-deficient MEFs treated with or without rapamycin. Enlarged images of the yellow box were indicated on the right side. Images were detected by laser confocal-scanning microscopy. Bar, 10 μm. **d** Quantification of the number of LC3 and LAMP2 colocalized puncta per cell. Data represent mean ± SEM; *n* = 3. **P < 0.01; ***P < 0.001.

To further determine whether DDRGK1 loss-induced inhibition of autophagic degradation was correlated with impaired autophagic flux, we transfected MEFs with a tandem-tagged mCherry-EGFP-LC3 plasmid, which exhibits yellow fluorescence in autophagosomes due to the combination of mCherry and EGFP signals. However, when autophagosomes are fused with lysosomes, the EGFP signal is quenched due to the acidic environment of lysosomes and only shows the mCherry signal^38,39^. Compared with the control group, DDRGK1 loss led to a significant increase of yellow puncta, while the only mCherry puncta that represent autolysosomes were severely decreased (Fig. 5a, b), suggesting that DDRGK1 deficiency causes an impaired autophagic flux in MEFs.

To more directly visualize the fusion of autophagosomes and lysosomes, we assessed the immunofluorescence colocalization of LC3 and LAMP2 proteins. Rapamycin was added to induce autophagy level, as well as autophagy flux. Compared with the EtOH group, both rapamycin and 4-OHT treatment induced a dramatic accumulation of autophagosomes. However, the colocalization of LC3 and LAMP2 in the rapamycin-treated group was robustly enhanced, while that in the 4-OHT group was dramatically decreased (Fig. 5c, d). These data indicate that DDRGK1 loss blocked the fusion of autophagosomes and lysosomes, thus leading to an impaired autophagic degradation.

### Result 6: DDRGK1 deficiency-induced apoptosis is associated with autophagy defect in MEFs

**Fig. 6:**
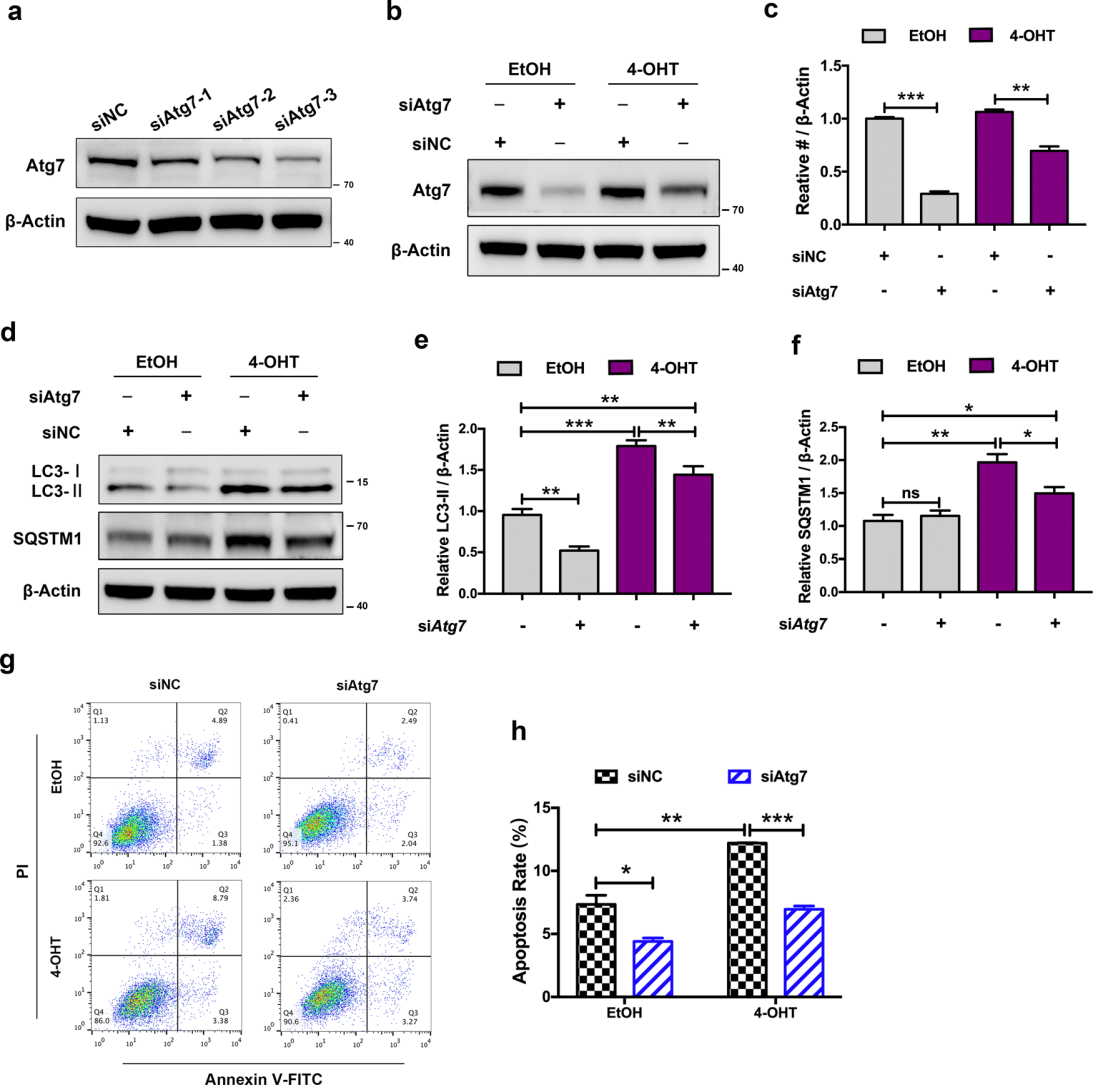
DDRGK1 loss-induced apoptosis is implicated with compromised autophagosome clearance. **a** MEFs were transfected with siNC oligos, si*Atg7*-1, si*Atg7*-2, and si*Atg7-3* (selected for the following experiments and hereafter referred to as si*Atg7*) siRNAs for 48 h, respectively. Cell lysates were collected to determine the protein levels of Atg7 by western blot. **b** Western blot for Atg7 protein from EtOH- or 4-OHT-treated MEFs transfected with or without siAtg7 for 48 h. **c** Quantification of Atg7 levels normalized to β-actin levels using densitometric analysis. Data represent mean ± SEM; *n* = 3. **P < 0.01; ***P < 0.001. **d** Western blot analysis of LC3 and SQSTM1 proteins in EtOH- and 4-OHT-treated MEFs transfected with or without siAtg7 for 48 h. **e**, **f** Quantification of LC3 and SQSTM1 levels normalized to β-actin level. Data represent mean ± SEM; *n* = 3. *P < 0.05; **P < 0.01; ***P < 0.001; ns not significant, *P* > 0.05. **g** Flow cytometry analysis for apoptosis in EtOH- and 4-OHT-treated MEFs transfected with or without si*Atg7* for 48 h. **h** Quantification of apoptosis rate in EtOH- and 4-OHT-treated MEFs. Data represent mean ± SEM; *n* = 3. *P < 0.05; **P < 0.01; ***P < 0.001.

To determine whether DDRGK1 deficiency-induced apoptosis was related to autophagy defect, we tested the apoptosis level in MEFs after si*Atg7* transfection. Firstly, we designed three small interfering RNAs (siRNAs) targeting *Atg7* and selected the one exhibiting the highest knockdown efficiency, si*Atg7*-3, for subsequent experiments (Fig. 6a). As is shown in Fig. 6b, c, Atg7 protein expression was significant decreased both in control and DDRGK1-deleted MEFs when transfected with *Atg7* siRNA. Moreover, Atg7 knockdown also significantly suppressed the accumulation of autophagosomes, as indicated with a remarkable decrease of LC3 protein in both control and DDRGK1-deleted groups, and a remarkable decrease of SQSTM1 protein in DDRGK1-deleted groups (Fig. 6d–f).

Flow cytometry analysis showed that loss of DDRGK1 significantly increased the apoptosis rate in MEFs (Fig. 6g, h). Notably, the enhanced apoptosis rate in DDRGK1-deleted MEFs was remarkably alleviated by si*Atg7* transfection, though apoptosis level was also decreased in EtOH-treated MEFs, probably because there was a certain degree of basal autophagy activity (Fig. 6g, h). These data suggest that DDRGK1 deficiency-induced apoptosis in MEFs is associated with aberrant accumulation of autophagosomes in some way.

### Result 7: DDRGK1 deficiency leads to dysfunction in lysosomes

**Fig. 7:**
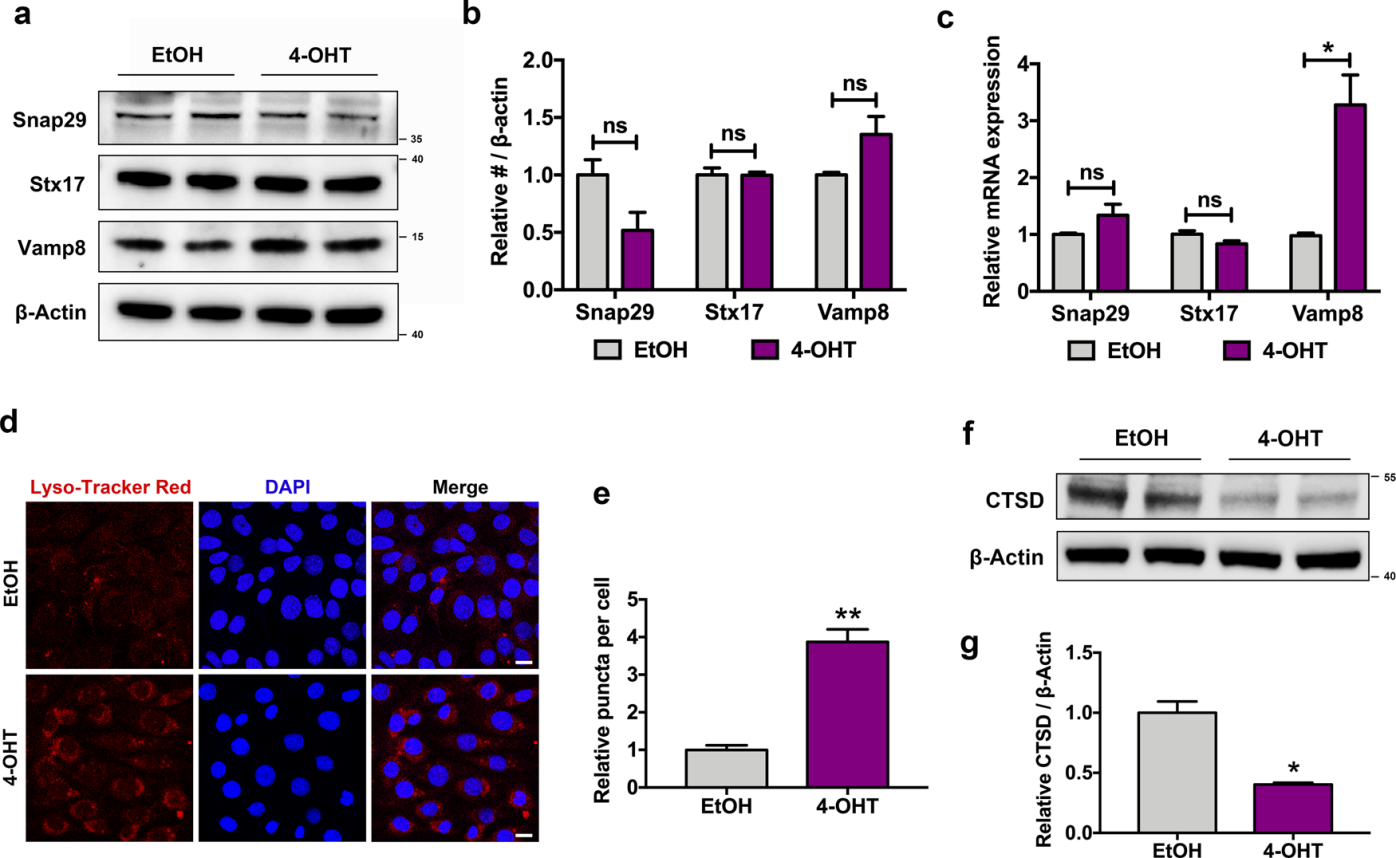
DDRGK1 deficiency results in lysosomal dysfunction. **a** Western blot analysis for SNAP29, Stx17, and VAMP8 proteins in EtOH- and 4-OHT-treated MEFs. **b** Quantitative expressions of SNAP29, Stx17, and VAMP8 proteins normalized to β-actin. Data represent mean ± SEM; *n* = 3. ns not significant, *P* > 0.05. **c** The transcriptional levels of *Snap29*, *Stx17*, and *Vamp8* in EtOH- and 4-OHT-induced MEFs. Data represent mean ± SEM; *n* = 3. *P < 0.05; ns not significant, *P* > 0.05. **d** Lyso-Tracker Red signals in control and DDRGK1-deleted MEFs. MEFs cultured in EtOH or 4-OHT for 4 days were then incubated with diluted Lyso-Tracker Red dye (diluted in cell culture medium in a ratio of 1:20,000) for another 20 min. Images were detected by laser confocal-scanning microscopy. Bar, 20 μm. **e** Quantification of normalized number of Lyso-Tracker Red puncta. Data were shown as mean ± SEM; *n* = 3. **P < 0.01. **f** Western blot analysis for CTSD expression in EtOH- and 4-OHT-induced MEFs. **g** Quantification of CTSD level normalized to β-actin expression. Data represent mean ± SEM; *n* = 3. *P < 0.05.

The fusion of autophagosomes and lysosomes is a complex dynamic process conducted by various factors, including proper acidic environment, normal function of lysosomal enzyme, and SNAREs. To investigate the specific mechanism of how autophagosome–lysosome fusion is suppressed, we detected the above factors separately. The results displayed that DDRGK1 loss increased the VAMP8 protein level without altering the level of Stx17 and SNAP29 proteins (Fig. 7a, b), which was consistent with the transcriptional level (Fig. 7c). Moreover, lysosomal acidification was detected by using the Lyso-Tracker Red probe, a lysosomal-specific fluorescent probe that contains a weakly basic group so that it can selectively stay in acidic lysosomes and exhibit red fluorescence. To our surprise, DDRGK1 loss significantly increased the fluorescence signals, suggesting the lysosomal pH was reduced rather than alkalinized (Fig. 7d, e). However, the deletion of DDRGK1 led to a compromised expression of CTSD (Fig. 7f, g), which was consistent with our proteomics results. Taken together, these data suggest that DDRGK1 loss leads to an impairment in CTSD level and lysosomal function.

### Result 8: DDRGK1 is involved in proteasome-mediated degradation of v-ATPase subunits

**Fig. 8:**
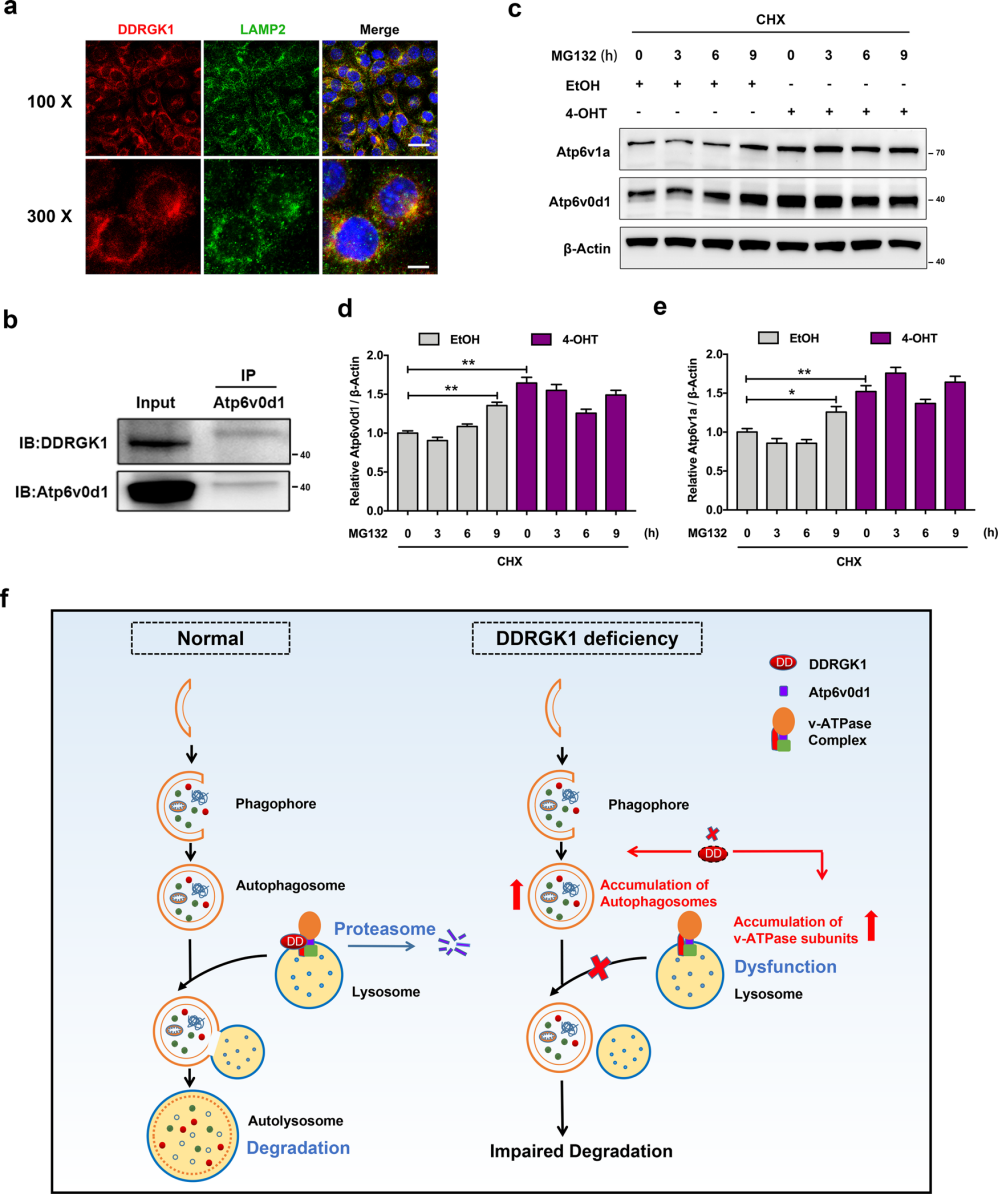
DDRGK1 is involved in proteasome-mediated degradation of v-ATPase subunits. **a** Colocalization of endogenous DDRGK1 and LAMP2 proteins in MEFs. Magnification is shown on the left side. Bar (upper), 40 μm; bar (lower), 10 μm. **b** Co-immunoprecipitation of DDRGK1 and Atp6v0d1 in MEFs without any treatment. Total cell lysates (input) were collected for immunoprecipitation with anti-Atp6v0d1, followed by immunoblotting with anti-DDRGK1. IP indicates the immunoprecipitation antibody; IB indicates the immunoblotting antibody. **c** Western blot for Atp6v0d1 and Atp6v1a proteins. MEFs were treated with EtOH or 4-OHT for 4 days. Before harvested, MEFs were pretreated with 20 μg/mL cycloheximide (CHX) for 12 h accompanied by 2 μM MG132 treatment for the indicated times. Cell lysates were then collected for western blotting. **d**, **e** Quantification of Atp6v0d1 (**d**) and Atp6v1a (**e**) levels normalized to β-actin levels. Data represent mean ± SEM; *n* = 3. *P < 0.05; **P < 0.01. **f** Schematic model for regulation of DDRGK1 on autophagy. Under normal conditions, DDRGK1 regulates ubiquitin–proteasome-mediated degradation of v-ATPase subunits (including Atp6v0d1 and Atp6v1a) and maintains the stable expression of them, which leads to a normal function of lysosomes, promotes the fusion of autophagosomes and lysosomes, and the subsequent autophagic degradation. However, DDRGK1 deficiency induces an aberrant accumulation of Atp6vd1 and Atp6v1a due to the impaired ubiquitin–proteasome degradation pathway, thus resulting in dysregulation of lysosome, blocked fusion of autophagosomes and lysosomes, and suppressed autophagic degradation.

To investigate the mechanism by which DDRGK1 affects lysosomal function, we then assessed the relationship between DDRGK1 and lysosome. We thus examined the subcellular localization of DDRGK1, and found a remarkable colocalization of DDRGK1 and LAMP2 (Fig. 8a), indicating DDRGK1 might be localized on the lysosomal surface. A previous study of Mümine Şentürk et al.^40^ has reported that ubiquilin, a ubiquitin-binding protein, mediates lysosome acidification by interacting with subunits of lysosomal proton pump, v-ATPase, thus facilitating autophagic flux. Considering the presence of v-ATPase subunits on our LC–MS data, we wonder if they have an interaction with DDRGK1. The co-immunoprecipitation assay showed that DDRGK1 indeed interact with Atp6v0d1 (Fig. 8b). To determine whether the enhancement of v-ATPase subunits during DDRGK1 deficiency was correlated with proteasome-dependent degradation, we performed MG132 incubation for different period of time in cells that pretreated with cycloheximide (CHX, an inhibitor of protein synthesis). Interestingly, MG132 treatment induced an accumulation of Atp6v0d1 and Atp6v1a in control MEFs, whereas such accumulation was not observed in DDRGK1-deficient cells (Fig. 8c–e). Especially after 9 h treatment with MG132, both Atp6v0d1 and Atp6v1a increased to a level similar to that of the DDRGK1-deleted groups (Fig. 8c–e), indicating that DDRGK1 accelerates proteasome-mediated degradation of v-ATPase subunits.

## Discussion

In the present study, we show that DDRGK1 deficiency in MEFs leads to an aberrant accumulation of autophagosomes due to the enhanced autophagy induction (as indicated with activation of mTORC1 signaling) and impaired autophagic degradation (as indicated with impaired autophagic flux), which ultimately aggravates apoptosis in MEFs. Furthermore, DDRGK1 is required for maintaining a proper expression of v-ATPase subunits by modulating ubiquitin–proteasome degradation pathway, as its deficiency induces aberrant accumulation of Atp6vod1 and Atp6v1a, which might be a trigger for lysosomal dysfunction and impaired autophagic flux. Taken together, our findings demonstrate an important role of DDRGK1 as an autophagy regulator by controlling lysosomal function, providing a novel insight into the molecular and cellular mechanism of DDRGK1 function.

The MEFs in our study were harvested from DDRGK1^F/F^:ROSA26-CreERT2 mice, so its DDRGK1 depletion can be efficiently induced by 4-OHT treatment. This makes it a perfect model to investigate the mechanism of DDRGK1. First, DDRGK1 seems to participate in autophagy process as we identified an abundant number of differentially expressed proteins enriched in lysosome- and autophagy-related pathways, during DDRGK1 deletion. This hypothesis was then confirmed by the enhanced accumulation of autophagosomes in DDRGK1-deleted MEFs. N’Diaye et al. argue that loss of UBQLN1 and 2 (two ubiquitin-binding proteins) causes a dual effect on autophagy, with increased autophagosome formation on the one hand, but decreased delivery of autophagosomes to lysosomes on the other hand^41^. Similarly, our data also validate a dual effect of DDRGK1 deficiency on autophagy, showing enhanced autophagy initiation by inactivating mTOR and activating AMPK kinase, but suppressed autophagic degradation with impaired autophagy flux. Based on these data, we hypothesize that, the impaired autophagic turnover induced by DDRGK1 loss may causes physiological imbalance in MEFs.

It is reported that excessive accumulation of autophagosomes induces apoptosis and cell death^42,43^. Indeed, in our study, the dual effect of DDRGK1 loss on autophagy aggravates autophagosomes accumulation and apoptosis, which could be attenuated by si*Atg7* transfection (serves as a way to block autophagy induction). Interestingly, Atg7 knockdown also alleviated the apoptosis in EtOH-treated groups, although the decline was lower than that of the DDRGK1-deleted groups, probably because of a certain degree of basal autophagy level in MEFs.

As autophagic degradation is closely related to autophagosome–lysosome fusion and lysosomal function^44,45^, we analyzed the function of lysosome during DDRGK1 loss. Though there was no significant change on STX17, SNAP29, and VAMP8 protein levels in our data, the interactions of them still remains to be verified in the future researches. Instead, the significantly decreased CTSD protein during DDRGK1 loss indicates a decreased lysosomal degradation capacity, which may in turn block the fusion of autophagosomes and lysosomes. In fact, reduced CTSD level has been demonstrated to impair the fusion of autophagosomes and lysosomes in the previous study^46^. Moreover, the aberrant accumulation of v-ATPase subunits also indicates a dysfunction in lysosomes. Given that v-ATPase is necessary for translocation of mTORC1 to lysosome surface, where mTORC1 is activated^47^, it is likely that the impaired mTORC1 activity is somewhat caused by v-ATPase dysfunction, which would explain why DDRGK1 deficiency has a dual effect of on autophagy regulation.

As DDRGK1 loss impairs lysosomal function, there might be a potential interaction between DDRGK1 and lysosome. To our surprise, we detected a colocalization of DDRGK1 with LAMP2, indicating a localization of DDRGK1 on lysosomal membrane or an interaction of DDRGK1 with lysosomal proteins. In fact, previous findings of Mümine Şentürk et al. have demonstrated an interaction between ubiquilin and v-ATPase, while ubiquilin loss impairs v-ATPase units levels and lysosomal acidification^40^. Consistently, DDRGK1 interacts with Atp6v0d1, one of the v-ATPase subunits presented in our proteomic data. Nevertheless, the acidification of lysosome was enhanced in our DDRGK1-deleted MEFs as indicated with an increased signal of Lyso-Tracker Red. Based on the contrary results, we speculate that lysosomes need to maintain an appropriate range of pH for normal function, either too high or too low may affect its degradation efficiency.

Recent studies show that DDRGK1 inhibits proteasomal degradation and is indispensable for maintaining the stability of related proteins, such as SOX9 (ref. ^48^) and IRE1α (ref. ^49^). Interestingly, in the present study, we found DDRGK1 is required for ubiquitin–proteasome-mediated v-ATPase subunits degradation, while DDRGK1 deletion blocked the proteasomal degradation, leading to elevated expression of Atp6v0d1 and Atp6v1a. This difference may be due to the different binding forms between DDRGK1 and target proteins. Therefore, we propose that DDRGK1 serves as a chaperone for v-ATPase units, and promotes normal lysosomal function by maintaining the stable expression of v-ATPase units.

In conclusion, our findings put forward a novel role of DDRGK1 in autophagy regulation via modulating lysosomal function, which provide a critical insight into DDRGK1 function. Thus, the study may provide a theoretical basis for the treatment strategies of various physiological diseases caused by DDRGK1 deficiency.

## Materials and methods

### Antibodies

Primary antibodies used in this study included DDRGK1 rabbit polyclonal antibody (Proteintech, 21445–1-AP, Wuhan, China); β-actin rabbit monoclonal antibody (Cell Signaling Technology, #4970, MA, USA); LC3B rabbit polyclonal antibody (Sigma, L7543, MO, USA); SQSTM1 mouse monoclonal antibody (Abcam, ab56416, Cambridge, UK); LAMP2 mouse monoclonal antibody (Santa Cruz, sc-18822, CA, USA); SNAP29 rabbit polyclonal antibody (Proteintech, 12704-1-AP); Stx17 rabbit polyclonal antibody (Proteintech, 17815-1-AP); VAMP8 rabbit polyclonal antibody (Proteintech, 15546-1-AP); Atg7 mouse monoclonal antibody (Proteintech, 67341-1-lg); Atp6v0d1 rabbit polyclonal antibody (Proteintech, 18274-1-AP); Atp6v1a rabbit polyclonal antibody (Proteintech, 17115-1-AP); CTSD rabbit polyclonal antibody (Proteintech, 21327-1-AP); 4E-BP1 rabbit monoclonal antibody (Cell Signaling Technology, #9644); phospho-4E-BP1 (Thr37/46) rabbit monoclonal antibody (Cell Signaling Technology, #2855); AMPK rabbit monoclonal antibody (Cell Signaling Technology, #5832); phospho-AMPKα (Thr172) rabbit monoclonal antibody (Cell Signaling Technology, #2535); and phospho-ULK1 (Ser757) rabbit monoclonal antibody (Cell Signaling Technology, #4202).

HRP-conjugated secondary antibodies, goat anti-mouse IgG (Abcam, ab6789) and goat anti-rabbit IgG (Abcam, ab6721), were all purchased from Abcam. Fluorophore-conjugated secondary antibodies, TRITC-conjugated goat anti-rabbit IgG (H + L; ZF-0316) and Alexa Fluor 488-conjugated goat anti-mouse IgG (H + L; ZF-0512) were purchased from Zhongshan Golden Bridge Biotechnology (Beijing, China).

### Animals, tissues, cell culture, and treatment

All procedures with mouse were conducted in accordance with the guidelines of the Animal Research Institute Committee at Nanjing Agricultural University (SYXK-2017-0027). Immortalized MEFs were derived from the DDRGK1^F/F^:ROSA26-CreERT2 mice. Briefly, harvest mouse embryos (genotyping, DDRGK1^F/F^:ROSA26-Cre/ERT2^+/+^ or DDRGK1^F/F^:ROSA26-Cre/ERT2^+/−^) at E13 or E14. Dissect head and red organs of embryos, wash in PBS and place all embryos in a clean Petri dish. Finely mince the tissue using a sterile razor blade until it becomes possible to pipette. Add 1 mL of 0.05% trypsin/EDTA (Gibco, NY, USA). After each 5 min of incubation, dissociate cells by pipetting up and down thoroughly. The fibroblasts are the only cells that have the ability to attach to the flasks with the DMEM medium (HyClone, Utah, USA). Ideally, cells are 80–90% confluent after 24 h and at this stage, then transfect with large T lentivirus, select the positive immortalized cells with puromycin after 2 days, expand the positive cells. We also generated DDRGK1 hepatocyte-specific knockout mice (DDRGK1^F/F^:Alb-Cre+) by crossing DDRGK1 floxed mice with Alb-cre^+^ mice (C57BL/6 background), and harvested liver tissues from mice at age of 8–12 weeks to investigate the relationship between DDRGK1 and autophagy.

The MEFs were cultured in DMEM medium supplemented with 10% fetal bovine serum (Sigma) and 50 μg/mL penicillin/streptomycin at 37 °C with 5% CO_2_. MEFs were treated with 4-OHT (2 μM, Sigma) or ethanol (EtOH) for 4 days to induce DDRGK1 deletion or as a control. Baf-A1 (10 nM, Selleck, S1413, TX, USA) was added to the indicated medium for 2 h before the MEFs were harvested. For autophagy induction, rapamycin (200 nM, Selleck, S1039) was supplemented to control and DDRGK1-deleted MEFs for 24 h. To test the stability of proteins, MEFs were pretreated with 20 μg/mL CHX (Sigma, A6185) for 12 h to inhibit protein synthesis, then incubated with 2 μM MG132 (Sigma, M7449) for the indicated times.

### RNA extraction and quantitative real-time PCR (qRT-PCR)

Total RNA was isolated from cultured MEFs using Trizol (Invitrogen, CA, USA). cDNA was synthesized using the PrimeScript^TM^ RT Master Mix (TAKARA, Shiga, Japan). qRT-PCR was performed using SYBR Green Master Mix (Vazyme, Nanjing, China) on a Step-One PCR system (Applied Biosystems, CA, USA). Primers used for the qRT-PCR were listed in the Supplementary Table S1 and all the expression data were normalized against *β-actin* as a control.

### Plasmid transfection and RNA interference

For transfection of DNA plasmid, recombinant mCherry-EGFP-LC3 plasmid was transfected into cells using Lipofectamine 3000 reagent (Invitrogen), according to the manufacturer’s instruction. Approximately 1 μg of plasmid with 2 μL P3000 reagent and 2 μL of Lipofectamine 3000 reagent (according to 12-well plate) were separately added into 50 μL of Opti-MEM (Gibco) medium, then mixed together for 10 min at room temperature (RT) and added to cells in culture medium. For RNA interference, MEFs were transfected with Atg7 siRNA or negative control siRNA (GenePharma, Shanghai, China; see Supplementary Table S2 for sequences) via Lipofectamine RNAiMAX reagent (Invitrogen), according to the manufacturer’s instructions. Briefly, ~50 pmol of siRNA and 3 μL of Lipofectamine RNAiMAX reagent (according to 12-well plate) were separately added into 50 μL of Opti-MEM medium, then mixed together for 5 min and transfected into MEFs.

### Immunoprecipitation and western blotting

The cells or tissues were lysed in IP lysis buffer or RIPA lysis buffer (Beyotime, Beijing, China) containing a complete protease inhibitor cocktail (Roche, Basel, Switzerland). Whole-cell lysates were centrifuged with 12,000 × *g* for 10 min at 4 °C and the supernatant were used for immunoprecipitation via incubation with Atp6v0d1 antibody at 4 °C overnight, followed by incubation with Pierce Protein A/G Magnetic Beads (Thermo Fisher Scientific, 88802, MA, USA) for 1 h to affiliate the antigen sample/antibody mixture. The magnetic beads were then washed three times with IP buffer and one time with purified water. After being eluted, immunoprecipitates were boiled in SDS loading buffer for 5 min at 95 °C and separated by SDS–PAGE Gel (Genscript, Nanjing, China), and transferred onto polyvinylidene fluoride membrane (Millipore, MA, USA). Western blot experiments were performed with the indicated antibodies and visualized by WesternBright^TM^ ECL kit (Advansta, CA, USA).

### Flow cytometry with Annexin V-FITC/PI staining

Flow cytometry analysis using the Annexin V-FITC/PI Apoptosis Detection Kit (Vazyme) was performed, according to the manufacturer’s instruction. In brief, harvested MEFs were incubated with FITC and PI for 10 min at RT and sorted by FACScan Flow Cytometer (Becton Dickinson, NJ, USA) afterward. Apoptotic events were recorded as a combination of Annexin V+/PI− (early apoptotic) and Annexin V+/PI+ (late apoptotic) events, and results were displayed as percentage of annexin V-positive cells analyzed using FlowJo software. All experiments were repeated in triplicate independently.

### Immunofluorescent staining

MEFs seeded on coverslips in 12-well plates were fixed in 4% paraformaldehyde for 20 min at RT, then permeabilized by PBS with 0.5% Triton X-100 for 20 min at RT. MEFs were washed three times with PBS, then blocked with 5% BSA in PBS for 1 h at RT. Then, cells were incubated with anti-LAMP2 mouse antibody mixed with anti-LC3 or DDRGK1 rabbit antibodies at 4 °C overnight. After washing three times with PBS, cells were then incubated with TRITC-conjugated anti-rabbit IgG and Alexa Fluor 488-conjugated anti-mouse IgG antibody for 1 h, and counterstained with DAPI for 10 min. Finally, the coverslips were mounted on glass slides and observed under a laser-scanning confocal microscope (Carl Zeiss, Zeiss LSM 900, Jena, Germany) and images were photographed at random positions for each group.

### Detection of autophagic flux

MEFs were seeded on coverslips in 12-well plates and transfected with the mCherry-EGFP-LC3 plasmid for ~32 h. Then, the living cells were quickly imaged with Zeiss LSM 900.

### Lyso-Tracker Red staining

After 4-day treatment with EtOH or 4-OHT, MEFs seeded on coverslips in 12-well plates were then incubated with Lyso-Tracker Red (Beyotime) at a dilution of 1:20,000 for 15 min, and then counterstained with DAPI for 10 min. Images were photographed by Zeiss LSM 900.

### Transmission electron microscope

MEFs treated with EtOH or 4-OHT for 4 days were harvested by trypsin digestion and fixed with 2.5% glutaraldehyde for 24 h at 4 °C. Samples were then postfixed with 1% osmium tetroxide for 2 h at 4 °C, washed, and stained with 2% aqueous uranyl acetate for 2 h. Then, samples were dehydrated with sequential gradient ethanol, embedded in Epon812, sectioned to ~50 nm, and mounted on carbon-coated copper grids. Ultrathin sections were counterstained with uranyl acetate and lead citrate and observed, using a transmission electron microscope (JEOL, JEM-1010, Tokyo, Japan).

### Statistical analysis

Statistical analysis and graphs were performed and drawn with GraphPad Prism v.7 Software. Statistical significance was assessed by unpaired and two-tailed Student’s *t* test, and data were presented as means ± SEM of three independent experiments. *P < 0.05, **P < 0.01, and ***P < 0.001.

## Supplementary information

Supplementary Figure

Table S1

Table S2

Table S3

Table S4

Table S5

Table S6
