# Supplementary material for: DDRGK1, a crucial player of ufmylation system, is indispensable for autophagic degradation by regulating lysosomal function
Source: Cell Death Dis. 2021 Apr 20;12(5):416. doi: 10.1038/s41419-021-03694-9 (PMC8058061; doi:10.1038/s41419-021-03694-9)
Supplement: Supplementary file 1 — Supplementary Figure [file 41419_2021_3694_MOESM1_ESM.docx]

**Supplementary Figure 1**

**

**

**Fig S1. Proteomics and bioinformatics analyses of DDRGK1-deleted MEFs.** (a) Hierarchical clustering analysis of DDRGK1-dependent proteins expression changes in observations. The row cluster indicated similarity among proteins and the column cluster indicated similarity among samples. The color bar denoted protein expression change after logarithm analysis，the color changed from green to red with the value growed bigger. (b) Statistics of KEGG pathways of up- and down-regulated differential expressed proteins in 4-OHT- or EtOH-treated groups. The percentage of the proteins in each pathway is listed next to the corresponding section.
